# Gene clusters of *Hafnia alvei* strain FB1 important in survival and pathogenesis: a draft genome perspective

**DOI:** 10.1186/1757-4749-6-29

**Published:** 2014-07-15

**Authors:** Jia-Yi Tan, Wai-Fong Yin, Kok-Gan Chan

**Affiliations:** 1 Division of Genetics and Molecular Biology, Institute of Biological Sciences, Faculty of Science, University of Malaya, Kuala Lumpur 50603, Malaysia

**Keywords:** *Hafnia alvei*, Gut pathogen, Widespread Colonisation Island, *tad*, Ethanolamine utilisation, *eut*, Siderophore, Next generation sequencing

## Abstract

**Background:**

*Hafnia alvei* is an opportunistic pathogen involved in various types of nosocomical infections. The species has been found to inhabit food and mammalian guts. However, its status as an enteropathogen, and whether the food-inhabiting strains could be a source of gastrointestinal infection remains obscure. In this report we present a draft genome of *H. alvei* strain FB1 isolated from fish paste meatball, a food popular among Malaysian and Chinese populations. The data was generated on the Illumina MiSeq platform.

**Results:**

A comparative study was carried out on FB1 against two other previously sequenced *H. alvei* genomes. Several gene clusters putatively involved in survival and pathogenesis of *H. alvei* FB1 in food and gut environment were characterised in this study. These include the widespread colonisation island (WCI), the *tad* locus that is known to play an essential role in biofilm formation, a *eut* operon that might contribute to advantage in nutrient acquisition in gut environment, and genes responsible for siderophore production This features enable the bacteria to successful colonise in the host gut environment.

**Conclusion:**

With the whole genome data of *H. alvei* FB1 presented in this study, we hope to provide an insight into future studies on this candidate of enteropathogen by looking into the possible mechanisms employed to survive stresses and gain advantage in competitions, which eventually leads to successful colonisation and pathogenesis. This is to serve as the basis for more effective clinical diagnosis and treatment.

## Background

*Hafnia alvei* is a flagellated, motile, facultative anaerobic opportunistic pathogen of the *Enterobacteriaceae* family, which is also known to play a role in microbial food spoilage [1]. This species has been isolated from a wide range of nosocomical infections, including septicaemia, as well as respiratory, enteric, and urinary tract infections [2,3]. Apart from that, *H. alvei* has also been commonly found to be present in abundance within communities of *N*-acyl homoserine lactone (AHL)-producing food spoilers [1,4].

Although *H. alvei* has been known to inhabit gastrointestinal tracts of various animal species, its status as an enteropathogen remains disputable. Clinical cases associate with *H. alvei* have been most intensively reported in the 1990s. However, solid evidence supporting the fact of the species being the sole cause of gastrointestinal infection, and whether it could be acquired via food is yet to be found. Several groups have attempted to investigate the possible pathogenesis pathways of *H. alvei* via biochemical and *in vitro* approaches [5-7]. However, the molecular basis of the mechanisms has not yet been demonstrated.

In this study, we sequenced the genome of *H. alvei* FB1 isolated from fish paste meatballs, a food made of fish paste popular among Southern and overseas Chinese communities. The processes of mashing and mixing in the making of fish paste meatballs brought the ingredients into frequent contacts with food processing surfaces. Along the way, bacterial cells detached from the biofilm-contaminated surfaces could become entrapped and immobilised within the food matrices. *H. alvei* has been reported to be able to survive temperature as low as 0.2°C [8]. The ability of *H. alvei* to form biofilm [9] and the connection of the trait to the concentration of AHLs have made it an interesting subject of study in controlling chronic contamination in food industry [10]. It is of importance to find out that if this common microbial contaminant of food could also be a source of gut infection.

Advancement in the technology of next generation sequencing and availability of powerful bioinformatics pipelines enable bacterial genomes to be explored with much ease. To date, only two other *H. alvei* genomes have been sequenced, one being strain ATCC 51873, isolated from gut; and the other BIDMC 31, as a part of a study on carbapenem resistance, isolated from unspecified clinical source (http://www.ncbi.nlm.nih.gov/genome). Here we looked into the putative means of survival and pathogenesis of *H. alvei* as a candidate of food and gut pathogen in a comparative genomics perspective. This is hoped to provide an insight into the molecular diversity of the species through comparison between the strain originated from food and those from guts in order to provide a basis for more in depth investigation in the future.

## Methods

### Bacteria culture

*H. alvei* FB1 was among the four bacterial species isolated in April 2013 from a packet of vacuum-packed fish paste meatballs sold in local supermarket. The sample was spread on MacConkey agar (MAC) plates for selective and differential purposed. Single colonies were picked and sub-cultured for at least two times to ensure the purity of each isolate. The isolates were identified via Microflex MALDI Biotyper system (Bruker, Germany) and 16S rDNA PCR prior to sequencing. The identified strains were maintained routinely on Luria-Bertani (LB) agar plates (Scharlau, Germany) at 37°C.

### Genomic DNA extraction

Genomic DNA was extracted from overnight liquid culture with MasterPure™ DNA Purification Kit (Epicentre, USA) according to the protocol provided by the manufacturer. Routine quantification was performed on Qubit®2.0 Fluorometer with dsDNA High Sensitivity Assay Kit (Invitrogen, USA); whereas quality assessment with NanoDrop 2000 Spectrophotometer (Thermo Scientific, USA) and gel electrophoresis. DNA samples were normalised into concentration of 1.8 ng/μl prior to library preparation.

### Library preparation for genome sequencing

Sequencing template was prepared with Nextera DNA Sample Preparation Kit (Illumina, USA). Quality checking on the prepared library was performed using Agilent 2100 Bioanalyzer High Sensitivity DNA Kit (Agilent Technologies, Canada). Ten picomolar (10 pM) of denatured DNA library was loaded into the sequencing cartridge can sequenced on Illumina MiSeq platform.

### Assembly and annotation

Quality assessment, trimming and assembly of the sequencing reads were performed using CLC Genomic Workbench 6 (http://www.clcbio.com). Raw reads were trimmed at Phred 30 and *de novo* assembled into 39 contigs. Assembled sequences were then annotated using RAST (Rapid Annotation using Subsystem Technology) pipeline [11].

### Genome comparison and phylogenetic analysis

A whole-genome-based phylogenetic tree was constructed by means of Composition Vector Tree (CVTree) version 2 [12]; while a sequence-based genome comparison was performed with RAST. The choices of organisms to be included were made according to the list of ‘closest neighbours’ presented by RAST. The whole genome sequence (WGS) data was obtained from the NCBI database.

## Quality assurance

The 16S rDNA gene was extracted from the draft genome using RNAmmer 1.2 server [13]. A single copy was detected. A BLAST annotation against NCBI microbial 16S database has confirmed that it belongs to *H. alvei*.

## Initial findings

**Figure 1:**
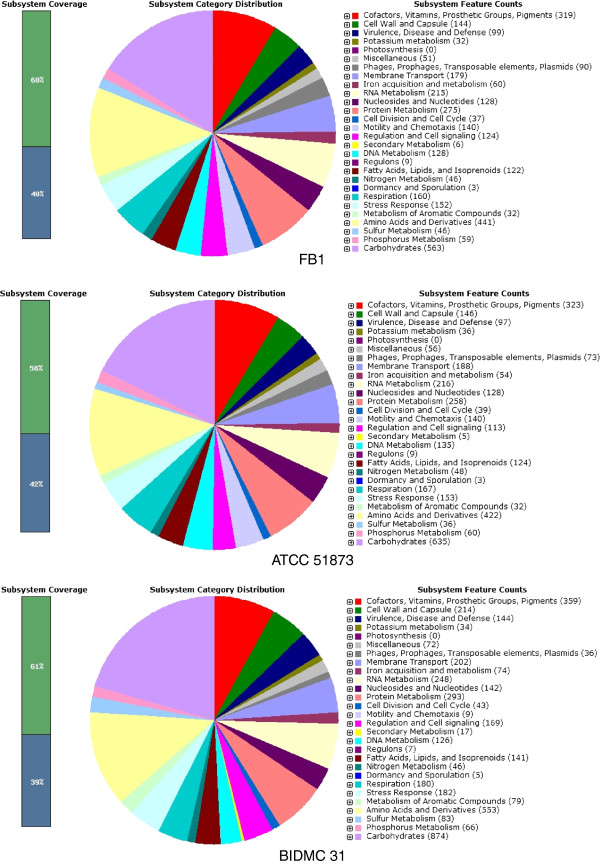
**Subsystem category distribution statistics for *H. alvei*.** The pie charts of three *H. alvei* strains are presented side by side to give an overview of the subsystem coverage and the counts of each subsystem feature.

The basic statistics of this draft genome are summarised in Table 1. Sixty percents of the 4,239 protein-coding sequences were categorised into 548 subsystems. Figure 1 is an overview of subsystem distribution in FB1 along with two other previously sequenced *H. alvei* genomes generated by RAST.

**Table 1:** List of genome statistics

**Attribute**	**Value**
Genome size	4,650,601 bps
No. of contigs	39
Minimum length of contigs	1,067 bps
Average coverage	119.1x
N50	256,161 bps
G + C content	48.90%
No. of subsystems	548
No. of RNAs	86
No. of CDS	4,239

**Figure 2:**
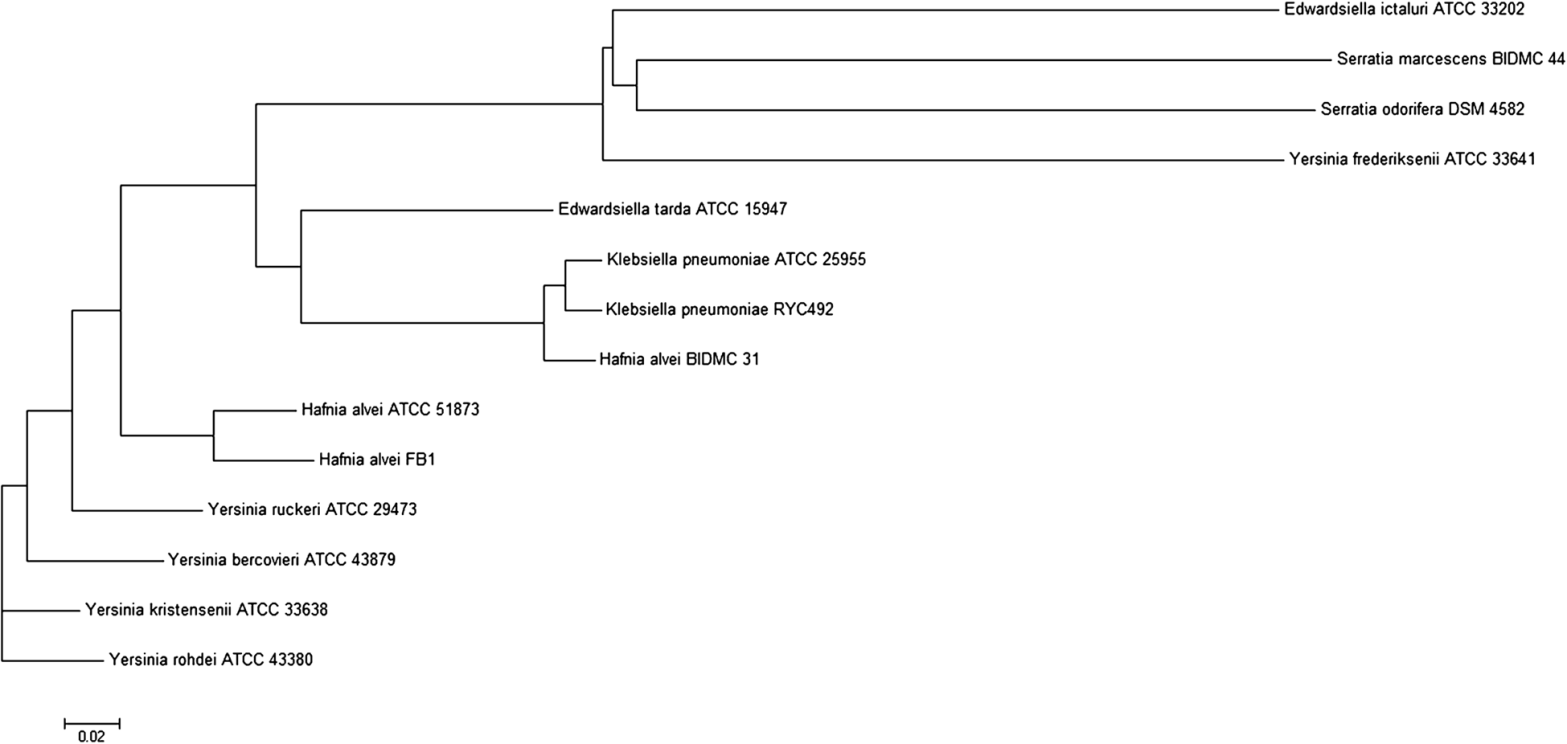
**Phylogenetic analysis on *H. alvei *FB1 genome.** The Neighbour-joining tree was constructed based on the whole genome DNA sequences with K-tuple length of 6.

The result of phylogenetic analysis, displayed as a Neighbour-joining tree in Figure 2, showed the probable evolutionary relatedness of three *H. alvei* strains and other selected organisms (genera *Edwardsiella*, *Serratia*, and *Yersinia* were among the closest neighbours presented by RAST). It was found that FB1 was most related to ATCC 51873; BIDMC 31 was, however, grouped closer to *Klebsiella pneumoniae* – a result consistent with that of 16S rDNA identification of the strain.

**Figure 3:**
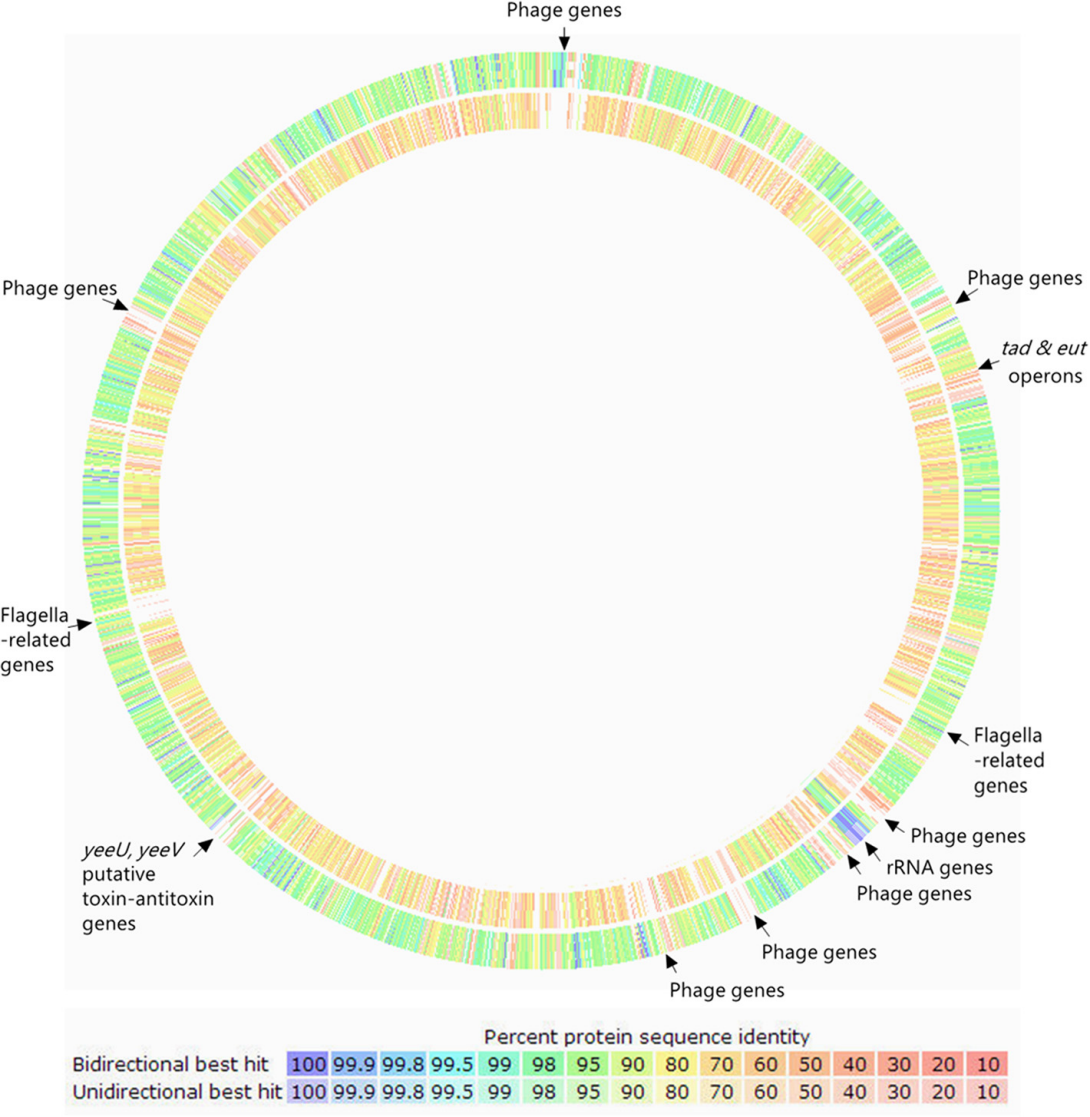
**Genome sequence comparison of *H. alvei *ATCC 51873 (outer) and *H. alvei *BIDMC 31 (inner).** Level of similarity is indicated by the intensity of colour. Regions containing sequences that do not have a match in the compared genomes were presented as ‘gaps’.

A sequence-based comparison between the two *H. alvei* genomes and FB1 was performed, and the result was presented as a colour-labelled circular map in Figure 3. It was shown that ATCC 51873 displayed a higher level of sequence similarity to FB1 (visualised in green). Gaps on the map indicated the presence of genes in FB1 genome that did not have a match in the compared genomes. The intra-species difference between FB1 and ATCC 51873 marked mainly by the presence of various phage-related genes. Two relatively large gaps observed in the BIDMC 31 circle reflected the lack of gene clusters involved in flagella biosynthesis. The genomic data strongly suggests that BIDMC 31 lacks motility – a trait that resembles *K. pneumoniae*. From Figure 1 we see that only nine out of 4,398 CDS covered in subsystems contribute to motility and chemotaxis in contrast to 140 in both FB1 and ATCC 51873.

**Figure 4:**
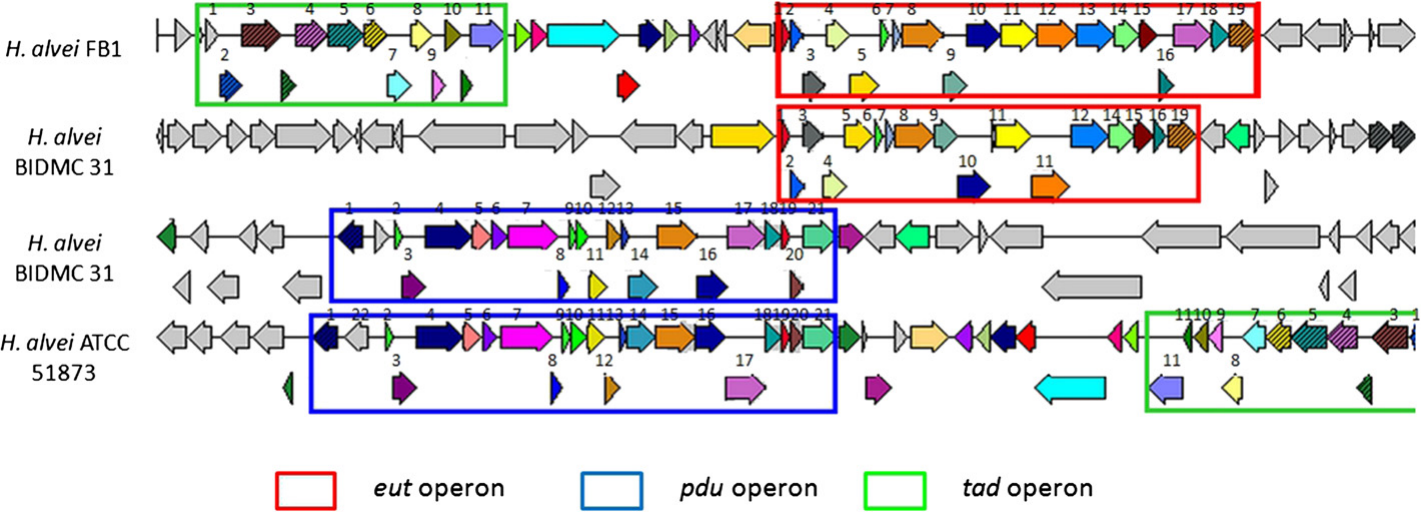
**Orientation of *tad*, *eut*, and *pdu *operons in *H. alvei *FB1, ATCC 51873, and BIDMC 31.***tad* operon: 1. Type IV prepilin peptidase TadV/CpaA; 2. Flp pilus assembly protein RcpC/CpaB; 3. Type II/IV secretion system secretin RcpA/CpaC; 4. Type II/IV secretion system ATPase TadZ/CpaE; 5. Type II/IV secretion system hydrolase TadA/VirB/CpaF; 6. Flp pilus assembly protein TadB; 7. Type II/IV secretion system protein TadC; 8. Flp pilus assembly protein TadD; 9. Flp pilus assembly membrane protein TadE; 10. Flp pilus assembly surface protein TadF; 11. Flp pilus assembly protein TadG. *eut* operon: 1. Ethanolamine utilisation polyhedral –body-like protein EutS; 2. Ethanolamine utilisation protein EutP; 3. Ethanolamine utilisation protein EutQ; 4. ATP:Cob(I)alamin adenosyltransferase; 5. Phosphate acetyltransferase; 6. Ethanolamine utilisation polyhedral-body-like protein EutM; 7. Ethanolamine utilisation polyhedral-body-like protein EutN; 8. Acetaldehyde dehydrogenase; 9. Ethanolamine utilisation protein EutJ; 10. Ethanolamine utilisation protein EutG; 11. Ethanolamine permease; 12. Ethanolamine utilisation protein EutA; 13. Ethanolamine ammonia-lyase heavy chain EutB; 14. Ethanolamine ammonia-lyase light chain EutC; 15.Ethanolamine utilisation polyhedral-body-like protein EutL; 16. Ethanolamine utilisation polyhedral-body-like protein EutK; 17. Cob(III)alamin reductase; 18. PduT; 19. Ethanolamine operon regulatory protein EutR. *pdu* operon: 1. Propanediol utilisation transcriptional activator; 2. Propanediol utilisation polyhedral-body-like protein PduA; 3. Propanediol utilisation polyhedral-body-like protein PduB; 4. Propanediol dehydratase large subunit; 5. Propanediol dehydratase medium subunit; 6. Propanediol dehydratase small subunit; 7. Propanediol dehydratase reactivation factor large subunit; 8. Propanediol dehydratase reactivation factor small subunit; 9. Propanediol utilisation polyhedral-body-like protein PduJ; 10. Propanediol utilisation polyhedral-body-like protein PduK; 11. Propanediol utilisation protein PduL; 12. Propanediol utilisation protein PduM; 13. Propanediol utilisation polyhedral-body-like protein PduN; 14. ATP: Cob(I)alamin adenosyltransferase; 15. CoA-acylating propionaldehyde dehydrogenase; 16. Putative iron-containing NADPH-dependent propanol dehydrogenase; 17. Cob(III)alamin reductase; 18. Propanediol utilisation polyhedral-body-like protein PduT; 19. Propanediol utilisation polyhedral-body-like protein PduU; 20. Propanediol utilisation protein PduV; 21. Propionate kinase; 22. Propanediol diffusion facilitator.

The Widespread Colonisation Island (WCI) was found to present in both FB1 and gut-inhabiting ATCC 51873 but absent in BIDMC 31. The *tad* (*t*ight *ad*hererence) genes that make up the Widespread Colonisation Island (WCI) play an essential role in biofilm formation, colonization and pathogenesis in a number of genera of bacteria and archea through the formation of Flp (fimbrial low-molecular-weight protein) pili for adherence [14]. The ability of bacteria to form biofilm has been known to provide them with resistance against physical, chemical, as well as the gastric stress, of which their planktonic counterparts lack [15]. In this draft genome, the 12 *tad* genes necessary in forming all the adherence related phenotypes were found in Contig 18 (Figure 4). Similar orientations were also observed in ATCC 51873.

Our data also shows that Contig 18 of *H. alvei* FB1 draft genome contained an *E*thanolamine *Ut*ilisation (*eut*) operon that possibly contributes to the thriving of *H. alvei* in the gastrointestinal environment. The *eut* operon provides the bacteria their ability to utilise ethanolamine, a form of molecules present in abundance in host intestine, as a sole source of energy. The one found in *H. alvei* FB1 is the typical long operon of the *Enterobacteriaceae* family (Figure 4). The presence of a *eut* operon guarantees the successful survival and colonisation of *H. alvei* FB1 in the intestinal environment. There are some hypotheses suggesting that the presence of *eut* operon indicated a role in pathogenesis, as the breakdown of phosphoethanolamine in the epithelial cell membranes could disrupt normal gut functions [16]. Interestingly, sequence-based comparison performed showed that this operon was also found in BIDMC 31, but not ATCC 51873. Instead, the latter possessed a paralogous propanediol utilisation (*pdu*) operon, which is also present in BIDMC 31 on a separate contig. The functions of both operons involve formation of proteinaceous polyhedral microcompartments in which the entire metabolic processes, i.e., ethanolamine and propanediol metabolisms take place [17], and the sequence homologies were seen to present in the genes that contribute to the formation of polyhedral-body-like. Both operons have been reported to associate with survival and the expression of global virulence regulators based on a well studied example *Salmonella* Typhimurium [15].

Previous reports have suggested the role of horizontal gene transfer in the current distribution pattern of *tad* and *eut* operons across species. Phylogenetic analysis by Planet *et al.* revealed that horizontal gene transfer had been a common event along the evolutionary history of this gene cluster [16]; whereas Tsoy *et al*. have discussed the possibility of genes in *eut* and *pdu* operons being acquired separately from different origins [18]. The possibility of horizontal gene transfer and the diversity between closely related organisms suggest that these operons could be providing certain forms of selective advantage to the species. The close proximity of the two operons leads to the speculation on the possibility of a collective role of the operons in pathogenesis. More studies need to be done to explore the complicated evolutionary events that occurred.

**Figure 5:**
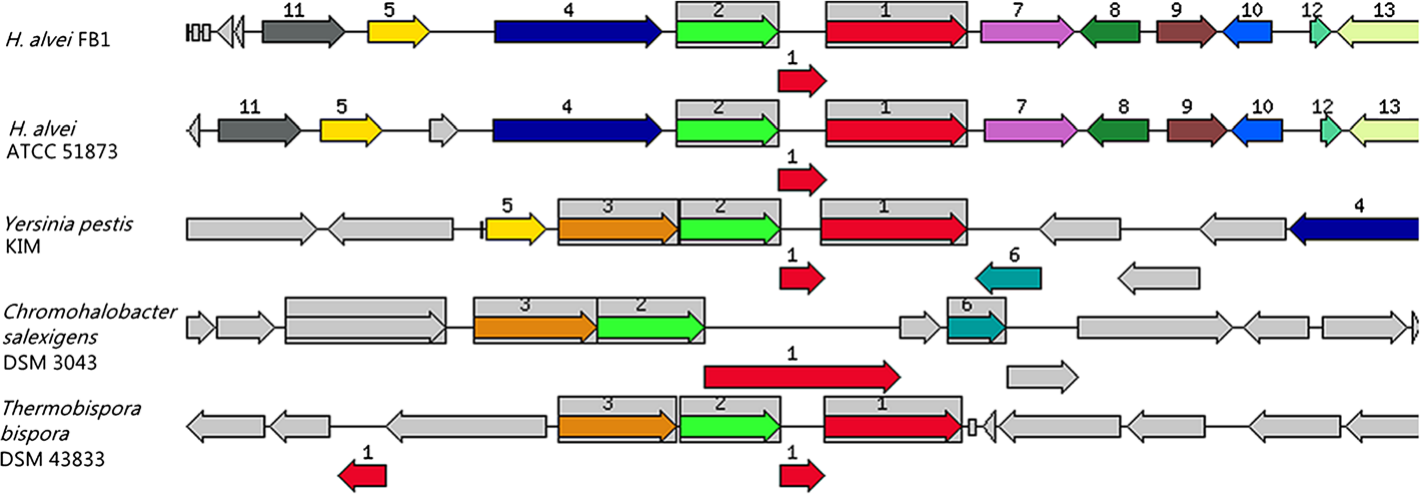
**Orientation of the siderophore gene cluster in *H. alvei *FB1 and that in closely related organisms.** 1(short): Desferrioxamine E biosynthesis protein, DesC, siderophore synthetase small component, acetyltransferase (AlcB homologue); 1(long): Desferrioxamine E biosynthesis protein, DesD, siderophore synthetase component, ligase (IucA, IucC homologue); 2: Siderophore [alcaligin-like] biosynthetic enzyme, monooxygenase (IucD homologue); 3: Siderophore [alcaligin-like] decarboxylase; 4: Ferrichrome-iron receptor; 5: Ferric reductase; 6: Iron-siderophore transport system, ATP-biding component; 7: Multidrug translocase MdfA; 8. YgiD; 9. ZupT; 10. 3,4-dihydroxy-2-butanone 4-phosphate synthase; 11. GGDEF domain protein; 12. Hypothetical protein; 13. Putative permease.

In addition to this, our genome analysis also showed the presence of genes important for iron uptake, another indication of virulence of *H. alvei*[19]*. H. alvei* has been reported to produce siderophore that is ‘neither aerobactin nor enterobactin’ by Podschun *et al.*[20] In Contig 6 of this draft genome we found a cluster of four genes possibly involves in the siderophore biosynthesis pathway similar to that of aerobactin (Figure 5). A complete set of *fhu* operon (*fhuABCD*) is also present, indicating that *H. alvei* FB1 is able to utilise the self-produced ferrochrome molecules as well as scavenge those available from the surroundings. The ability of siderophore production and uptake has been linked to virulence regulations and survival in an iron-deficient mammalian host environment [21].

## Future directions

The presences of the said gene clusters putatively secure *H. alvei* FB1’s way through the harsh environments towards the host gut and ensure its advantage in the interspecies competition in the gut environment. Differences were observed between the different strains of *H. alvei*. However, there was limited data available to perform a comparison that is able to show distinguished evolutionary tracks adapted by members of the species inhabiting different environments. With the genomic data available, our future study will be focusing on validation of the role of *tad* operon on biofilm formation and the presence of regulatory role of *eut* operon on the adherence trait of *H. alvei* via mutant, cell culture and transcriptomic approaches in order to gain a better understanding on the behavior of FB1 in response to stresses and changes in environment.

## Availability of supporting data

This whole genome shotgun project has been deposited at DDBJ/EMBL/GenBank under the accession JCKH01000000. The version described in this paper is version JCKH01000000.

## Abbreviations

AHL: *N-*acyl homoserine lactone; BLAST: Basic local alignment search tool; CDS: coding DNA sequence; LB: Luria-Bertani; RAST: Rapid annotation using subsystem technology; WCI: Widespread colonisation island.

## Competing interests

The authors declare that they have no competing interests.

## Authors’ contribution

JYT performed the DNA sequencing assay and data analysis. JYT, WFY and KGC contributed to the writing of the manuscript. All authors read and approved the final manuscript.
